# Draft genome sequence and phenotypic analysis of *Pseudozyma* sp. SY16 with evidence of binucleate cells

**DOI:** 10.3389/ffunb.2026.2001516

**Published:** 2026-10-29

**Authors:** Ami Rho, Dong-Yun Choi, Jin-Ho Yun, Youngshin Park, Hong Il Choi, Dae-Hyun Cho, Hee-Sik Kim, Yong Jae Lee

**Affiliations:** 1 Cell Factory Research Center, Korea Research Institute of Bioscience and Biotechnology, Daejeon, Republic of Korea; 2 Department of Bioresource & Environmental Engineering, University of Science and Technology, Daejeon, Republic of Korea

**Keywords:** mannosylerythritol lipids (MELs), PacBio-Illumina, Pseudozyma, Pseudozyma sp. SY16, whole genome sequence, yeast

## Abstract

Mannosylerythritol lipids (MELs) are glycolipid biosurfactants that have attracted considerable attention in cosmetic and industrial applications due to their favorable biocompatibility and stability. *Pseudozyma* sp. SY16 is a promising MEL-producing strain with high production capacity; however, the lack of genomic resources has limited our understanding of its genetic and cellular characteristics and hindered its application in metabolic engineering. In this study, we generated a high-quality draft genome of *Pseudozyma* sp. SY16 using a hybrid sequencing approach combining long- and short-read sequencing and comprehensively characterized its genomic and cellular features. The 20.2 Mb genome was assembled into 23 nuclear scaffolds and one mitochondrial scaffold, achieving a high BUSCO completeness of 98.9%. Comparative analysis of the MEL biosynthetic genes among closely related species within the family Ustilaginaceae revealed distinct patterns of gene conservation. The *mac1*, *mac2*, *mat1*, *mmf1*, and *emt1* genes were highly conserved in both gene content and genomic organization among SY16, *P. hubeiensis*, and *Mycosarcoma maydis*, whereas only *mmf1* was retained in the corresponding regions of *P. pruni* and *P. flocculosa*. This pattern suggests lineage-specific retention and loss of MEL biosynthetic genes during the evolution of family Ustilaginaceae. GenomeScope analysis revealed a predominantly single-peaked k-mer distribution, without a clear pattern indicative of either a haploid or diploid genome. Independently, confocal microscopy revealed frequent cells with two spatially separated DAPI-positive signals, consistent with an apparent binucleate morphology. Together, these findings establish a high-quality genomic resource for SY16 and provide new insights into MEL biosynthesis, genome evolution, and cellular organization, providing a foundation for future genetic and metabolic engineering of this non-model yeast toward enhanced MEL production.

## 1 Introduction

Biosurfactants are surface-active compounds of biological origin, primarily produced by microorganisms. Owing to their high biodegradability, low toxicity, and environmental sustainability, they have emerged as sustainable alternatives to conventional synthetic surfactants (Jimoh and Lin, 2019; Fernandes et al., 2023). Examples of naturally derived surfactants include animal-derived casein and lanolin, plant-derived lecithin and glucosides, and microbial biosurfactants such as sophorolipids, rhamnolipids, and mannosylerythritol lipids (MELs). However, animal-derived surfactants may raise ethical concerns related to animal use and potential competition with food resources. Plant-derived surfactants, although generally cost-effective and readily available from sources such as soybean and coconut, are susceptible to climatic and environmental conditions, which can affect the stability of raw material supply. In addition, variations in composition and functional properties among plant sources can make standardization of raw materials challenging. In contrast, microbial biosurfactants can be produced under controlled fermentation conditions, allowing greater control over production processes and more consistent product characteristics. Moreover, microbial producers can be readily modified through metabolic and genetic engineering to improve production efficiency and tailor product properties. These advantages have contributed to growing interest in microbial biosurfactants for a broad range of industrial and consumer applications.

Microbially produced biosurfactants are amphiphilic molecules composed of hydrophilic and hydrophobic moieties. The hydrophilic moiety typically consists of carbohydrates or amino acids, whereas the hydrophobic moiety is composed of saturated or unsaturated fatty acids (Banat et al., 2010). The amphiphilic nature of these molecules allows them to interact with both aqueous and lipid-rich environments, contributing to their surface activity and interactions with biological interfaces. In addition to their surface-active properties, the structural diversity and biodegradability endow them with favorable biocompatibility, including low cytotoxicity and reduced skin irritation compared with many conventional synthetic surfactants (Fei et al., 2020; Adu et al., 2023). Furthermore, many biosurfactants exhibit additional biological properties, including antimicrobial, moisturizing, and antioxidant activities (Banat et al., 2010; Karnwal et al., 2023), making them promising candidates not only as surfactants but also as functional ingredients in cosmetic and pharmaceutical formulations.

Among microbial biosurfactants, rhamnolipids, sophorolipids, and MELs are representative glycolipid biosurfactants that have been extensively investigated for their industrial potential. However, both rhamnolipids and acidic sophorolipids are anionic surfactants with physicochemical properties that can be affected by pH, and their anionic nature may cause skin irritation, limiting their use in cosmetic applications. In addition, rhamnolipid production has traditionally relied on *Pseudomonas aeruginosa*, an opportunistic pathogen, raising safety concerns for industrial production. Although sophorolipids generally show higher production yields than rhamnolipids, their potential for skin irritation remains a limitation. In contrast, MELs are nonionic glycolipids with favorable skin compatibility and good stability over a broad range of pH and temperature conditions. Their efficient production by non-pathogenic yeast under controlled fermentation conditions also offers advantages in terms of production safety and process control. Furthermore, the structural diversity of MELs allows their physicochemical properties to be tailored through changes in fatty acid composition and acetylation pattern, supporting their broad potential in cosmetic, pharmaceutical, and other industrial applications. Overall, MELs offer a favorable combination of stability, biocompatibility, and production potential.

Strains of the genus *Pseudozyma* are recognized as promising producers of the glycolipid biosurfactants MELs, and considerable efforts have been devoted to improving MEL production through fermentation optimization and metabolic engineering (Kim et al., 2006; Kunitake et al., 2019; Tran et al., 2022). A number of Ustilaginaceae yeasts have been identified as efficient MEL producers, including *Mycosarcoma maydis* (formerly *Ustilago maydis*), *Moesziomyces antarcticus* (formerly *Pseudozyma antarctica*), *Moesziomyces aphidis* (formerly *Pseudozyma aphidis*), *Moesziomyces rugulosa* (formerly *Pseudozyma rugulosa*), *P. tsukubaensis* and *Pseudozyma* sp. SY16 (Kim et al., 1999, 2002a, 2002b; Hewald et al., 2006; Kim et al., 2006; Fukuoka et al., 2007; Morita et al., 2007a, 2007b). Despite the identification of diverse MEL-producing yeasts and advances in production optimization, the industrial commercialization of MELs remains relatively limited, with only a small number of commercial production cases reported to date (Kim et al., 2006; Morita et al., 2010; Tran et al., 2022). Therefore, further characterization of MEL-producing yeasts and their genetic and metabolic features is important for improving MEL production and facilitating their broader industrial application.

Among MEL-producing yeast, *Pseudozyma* sp. SY16 has been reported to exhibit high MEL productivity, highlighting its potential as an industrial production host (Kim et al., 2006; Tran et al., 2022). However, despite its promising production characteristics, publicly available genomic resources for SY16 remain limited compared with those of extensively studied MEL-producing species such as *M. maydis* and *M. antarcticus*. Although genome assemblies of several *Pseudozyma* and related species have been reported, many remain fragmented or incompletely annotated, limiting their utility for comparative genomic and functional analyses. A high-quality and comprehensively annotated genome of SY16 is therefore needed to better characterize its genetic potential and facilitate the identification of genetic features associated with MEL biosynthesis and other industrially relevant traits.

Therefore, in this study, we generated a high-quality whole-genome assembly of *Pseudozyma* sp. SY16 using a hybrid assembly approach integrating long- and short-read sequencing. Comprehensive structural and functional genome annotation was subsequently performed to characterize the genomic features and genetic potential of SY16. Comparative genomic analyses were further conducted to examine the conservation and divergence of genes and genomic regions associated with MEL biosynthesis among related species. To further characterize the cellular and genomic features of SY16, confocal microscopy was used to examine its cellular morphology and nuclear organization, which was complemented by genome-wide analysis of heterozygosity. Together, these analyses provide a comprehensive genomic resource for *Pseudozyma* sp. SY16 and provide insights into its genetic and cellular characteristics, establishing a foundation for future studies on MEL biosynthesis, genetic manipulation, and metabolic engineering.

## 2 Materials and methods

### 2.1 DNA extraction and genome sequencing

*Pseudozyma* sp. SY16 was grown in a liquid YM medium (BD, Franklin Lakes, NJ, USA) at 30°C with shaking at 200 rpm. Genomic DNA was extracted using the FastDNA spin kit for soil (MP Biomedicals, CA, USA) according to the manufacturer’s instructions.

The whole genome was sequenced using PacBio Sequel and the Illumina HiSeq platform by Macrogen (Daejeon, South Korea). For PacBio sequencing, genomic DNA was converted into circular SMRTbell libraries and sequenced. The resulting subreads, which represent single-pass sequence of the insert DNA, were used for *de novo* genome assembly. For Illumina HiSeq sequencing, genomic DNA was randomly fragmented, followed by ligation of 5′ and 3′ sequencing adapters to construct paired-end libraries. The libraries were sequenced on the Illumina HiSeq platform using sequencing-by-synthesis (SBS) chemistry, and the resulting short-read data were used for genome polishing and error correction.

### 2.2 Genome assembly and annotation

*De novo* genome assembly was executed on the PacBio long-read sequencing data (~932× coverage; subread N50: 17,122 bp) using the Hierarchical Genome Assembly Process (HGAP). The initial assembly was polished using Quiver with PacBio reads. To maximize base-level precision, Illumina HiSeq short reads (~100× coverage after quality filtering; 2.0 Gb) were subsequently mapped to the polished genome for secondary error correction with Pilon v1.21, ultimately generating the high-accuracy consensus sequence.

Repetitive elements were identified and masked, and structural gene predictions were carried out using the MAKER v2.31.8 pipeline, incorporating ab initio gene predictions along with sequence alignment evidence. Functional annotation of predicted proteins was inferred using BLAST+ v2.7.1+ (E-value ≤ 10^-3^) against NCBI NR and NT databases.

Genome completeness was assessed using BUSCO v5.8.3 (Simão et al., 2015) against the basidiomycota_odb10 lineage dataset. The mitochondrial genome was identified and annotated using MitoHiFi v3.2.2 (Uliano-Silva et al., 2023) via Docker with the default contig-based approach (-c flag). The assembled nuclear genome contigs served as the input query, and the published mitochondrial genome of *Pseudozyma* sp. (*Pseudozyma* sp. mitochondrial genome (GenBank accession: MK714018.1; Han and An, 2019) was used as the reference sequence. Subsequently, the genome map of mitochondrial genomes was visualized using OGDRAW (Greiner et al., 2019).

### 2.3 k-mer-based genome profiling

Nuclear genome heterozygosity was estimated from 21-mer frequency distributions generated using Jellyfish v2.2.6 (Marçais and Kingsford, 2011). The resulting *k*-mer count histogram was analyzed using GenomeScope version 2.0 (Ranallo-Benavidez et al., 2020) to estimate genome size and heterozygosity and to characterize the overall *k*-mer distribution. These genomic profiles were subsequently evaluated together to investigate the ploidy and nuclear organization of the SY16 strain.

### 2.4 Functional gene annotation

Functional annotation included biosynthetic gene cluster (BGC) prediction via antibiotics and Secondary Metabolites Analysis Shell (antiSMASH) database v8.0.4 (Blin et al., 2025). Carbohydrate-active enzymes (CAZymes) were predicted using the dbCAN3 server (Zheng et al., 2023). Based on dbCAN3 guidelines, candidates identified by at least two of the three prediction algorithms – HMMER (E-value < 1e-15, coverage > 0.35), DIAMOND (E-value < 1e-102), and dbCAN-sub were retained. All bioinformatic workflows were executed within a Conda environment (Miniconda3, version 26.5.3; Anaconda Inc., Austin, TX). The assembled sequences generated in this study have been deposited in the NCBI database under accession numbers (JCARYC000000000).

### 2.5 Comparative genomic analysis

Orthology inference was performed using OrthoFinder v2.5.4 (Emms and Kelly, 2019). Single-copy orthologs shared across all target species were then individually aligned using MAFFT v7.525 (Katoh et al., 2019). The concatenated alignment was then used to construct a species tree using FastTree2 with the JTT+CAT model. To identify orthologous gene clusters, we downloaded high-quality genome data from closely related species within the family Ustilaginaceae.

Genome data for *P. flocculosa* PF-1, *P. hubeiensis* SY62, *P. pruni* CBS 10937, *P. tsukubaensis* NBRC 1940, *M. maydis* 521 were used for this analysis. Protein sequences predicted by Funannotate (Palmer and Stajich, 2020) for all yeast strains were used in this ortholog analysis. Orthologous analysis was performed via OrthoVenn3 (Sun et al., 2023) with the OrthoFinder algorithm, e-value cutoff of 1e-05 and inflation values of 1.5.

### 2.6 Homology and similarity analysis of MEL biosynthetic genes

To assess gene cluster organization and sequence similarity among MEL biosynthetic genes identified by BLASTP, the corresponding genomic regions were compared using Clinker v0.0.32 (Gilchrist and Chooi, 2021) through the CAGECAT server (Van Den Belt et al., 2023). Clinker performs pairwise protein sequence alignments between genes in the input clusters and generates interactive gene cluster comparison maps using clustermap.js.

### 2.7 Morphological and taxonomic characterization

For morphological analysis, specimens of *Pseudozyma* sp. SY16 were prepared for Scanning Electron Microscopy (SEM) and imaged using a SEM 515 (Philips) at an accelerating voltage of 20.0 kV. Nuclear behavior and cellular structure were visualized via confocal microscopy using a Leica STELLARIS WLL equipped with SpectraPlex (Leica, Wetzlar, Germany). For DAPI staining, 1 ml of cultured cells was harvested by centrifugation at 4,000xg for 3 min and the cells were then incubated with 0.1% Triton X-100 solution for 5 min at room temperature. Cells were then stained with 1 μg/ml DAPI (Invitrogen Corporation) for 10 min at room temperature in the dark, followed by two washing steps with 1xPBS. Confocal fluorescence images were captured using a 63×/1.40 oil immersion objective lens with a 1x optical zoom, maintaining the pinhole size at 1 AU at 580 nm emission.

For taxonomic identification, the Internal Transcribed Spacer (ITS) region was aligned using MAFFT v7.525 (Katoh et al., 2019). A maximum likelihood phylogenetic tree was constructed using IQ-TREE v3.1.2 (Wong et al., 2026) with automatic model selection.

## 3 Results

### 3.1 Cellular characteristics and dimorphic morphology of *Pseudozyma* sp. SY16

The *Pseudozyma* yeast strain SY16 was originally isolated from an oil-contaminated soil sample in Daejeon, South Korea, by employing the oil film-collapsing assay method as described by Kim et al. (1997). Strain SY16 exhibits robust growth in YM medium (**Figure 1A**). For MEL production (**Figure 1B**), the strain was cultivated in a specialized medium containing 21 g/L glycerin, 50 g/L oleic acid, 2.5 g/L K_2_HPO_4_, 2 g/L NH_4_NO_3_, 0.1 g/L NaH_2_PO_4_, 1 g/L yeast extract, 0.5 g/L MgSO_4_·7H_2_O, 0.1 g/L CaCl_2_·H_2_O, 0.02 g/L MnSO_4_·H_2_O. Under these optimized fermentation conditions, the wild-type strain achieved a MEL yield of 22 g/L (Tran et al., 2022).

**Figure 1:**
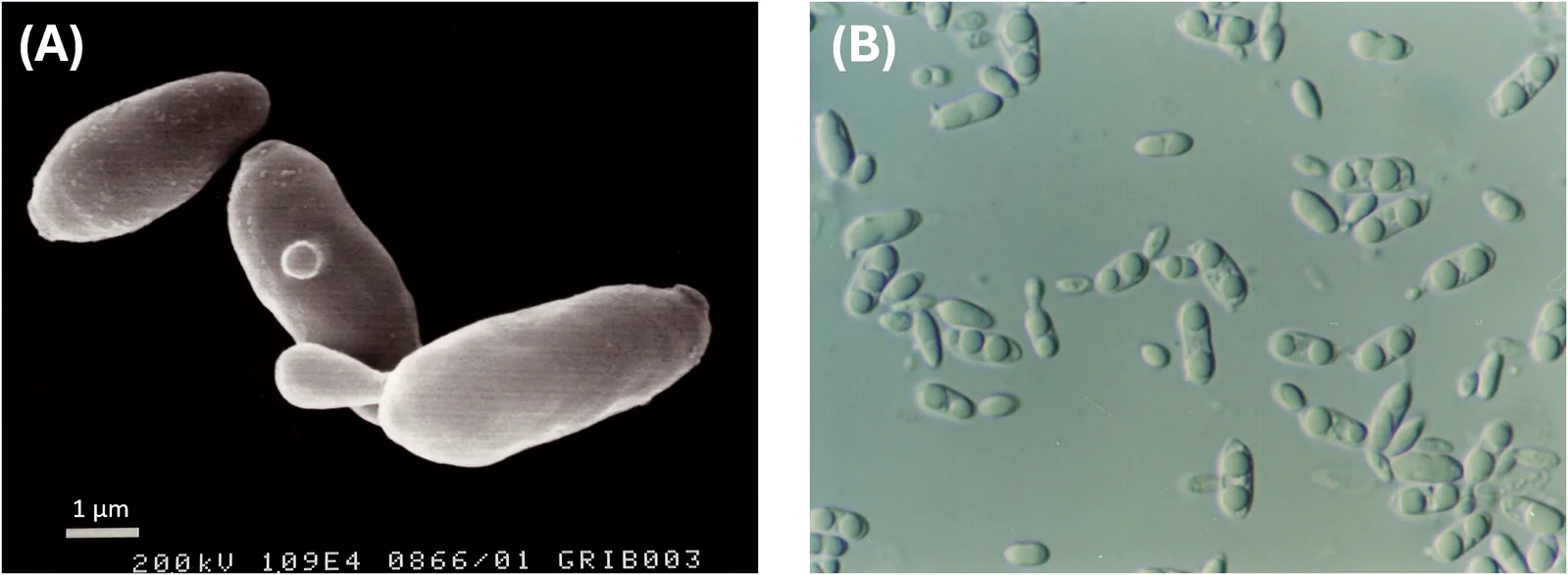
Microscopic observations of cellular morphology and MEL production in *Pseudozyma* sp. SY16. **(A)** SEM capturing the moment of cellular budding. **(B)** bright-field light micrograph of cells cultivated in a MEL-production medium; large intracellular circular bubbles represent the accumulated MELs within the cells.

During early-stage cultivation on YM agar plates, strain SY16 produced viscous, pale-yellow colonies (**Figure 2A**). Over time, pseudohyphal expansion was observed along the colony margins (**Figure 2B**). When these pseudohyphal regions were selectively streaked onto fresh media, the resulting growth exclusively retained the pseudohyphal morphology instead of reverting to the initial colony shape, indicating an irreversible phase transition (**Figure 2C**). Microscopic examination confirmed these macroscopic observations at the cellular level, revealing characteristic pseudohyphal morphology (**Figure 2D**). Specifically, daughter cells remained attached to mother cells post-budding while undergoing continuous polar elongation to form chain-like cellular structures. Together, these observations demonstrate that *Pseudozyma* sp. SY16 can undergo a distinct transition from yeast-like to pseudohyphal morphology, with the pseudohyphal phenotype being stably maintained upon subculturing.

**Figure 2:**
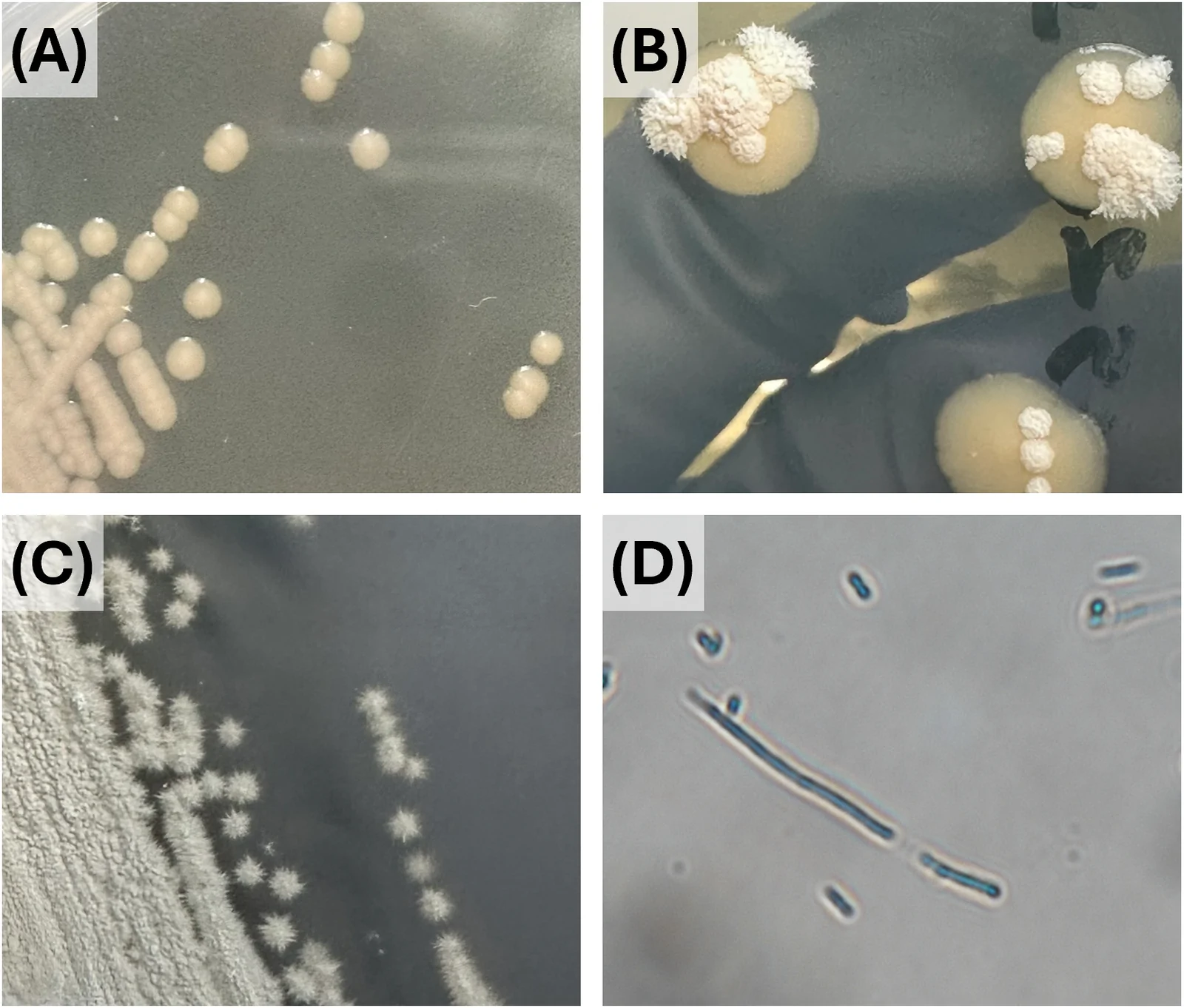
Morphological characteristics and isolation of pseudohyphae in *Pseudozyma* sp. SY16. **(A)** typical colony morphology of strain SY16. **(B)** development of pseudohyphae expanding from the colony over time. **(C)** uniform pseudohyphal growth after subculturing the isolated pseudohyphal region onto a new plate. **(D)** microscopic observation of pseudohyphal morphology.

### 3.2 Genome assembly and genomic features of SY16

A high-quality draft genome for strain *Pseudozyma* sp. SY16 was constructed with a total size of 20,223,705 bp (20.2 Mbp) distributed across only 23 nuclear scaffolds and 1 mitochondrial scaffold (**Table 1**). Notably, this represents a significant improvement in assembly continuity compared to the previously reported *P. tsukubaensis* NBRC 1940 genome, which stands at 23.8 Mbp but is highly fragmented into 12,840 scaffolds. The drastically reduced scaffold count in strain SY16 underscores the high-quality and near-complete nature of our hybrid assembly, providing a superior genetic reference for this species.

**Table 1:** The statistics of genome assembly for *Pseudozyma* sp. SY16 and related basidiomycetous yeast.

Species name	Strain	Size (Mb)	Nuclear Scaffolds	N50 (Kb)	GC content	Basidiomycota_odb10 (%) (C[S+D]/F/M)^a^	Genome sequencing technology	Accession number
*Pseudozyma* sp.	SY16	20.2	23	894.2	53.5%	98.9[98.8 + 0.1]/0.1/1.0	Illumina + PacBio	JCARYC000000000
*Pseudozyma tsukubaensis*	NBRC 1940	23.8	12,840	161.9	53.4%	98.7[98.6 + 0.1]/0.2/1.1	Illumina + Sanger	GCA_001736125.1
*Pseudozyma hubeiensis*	SY62	18.4	74	445.6	56.5%	97.8[97.8 + 0.1]/0.8/1.4	Illumina	GCF_000403515.1
*Pseudozyma flocculosa*	PF-1	23.3	39	919.9	65.5%	96.5[96.5 + 0.0]/0.8/2.7	454	GCF_000417875.1
*Pseudozyma pruni*	CBS 10937	17.9	112	319.2	57.8%	99.1[99.1 + 0.0]/0.2/0.7	Illumina	GCA_023212685.1
*Mycosarcoma maydis*	521	19.7	23	885.0	54.0%	98.9[98.8 + 0.1]/0.3/0.8	Sanger	Umaydis521_2.0

a C, complete; S, single-copy; D, duplicated; F, fragmented; M, missing.

The completeness of the assembled genome was further evaluated using BUSCO analysis against the basidiomycota_odb10 lineage dataset. The analysis revealed a high level of genome completeness, with 98.9% of the conserved BUSCO genes identified as complete, indicating that the genome assembly captured the vast majority of the expected gene content. Among the identified BUSCOs, 98.8% were present as single-copy genes, while only 0.1% were classified as duplicated. With only 0.1% fragmented and 1.0% missing genes, this indicates that nearly all conserved fungal genes were successfully recovered in the assembly.

To gain insight into the ploidy of *Pseudozyma* sp. SY16, we generated a *k*-mer profile (*k* = 21) and performed GenomeScope analysis (**Supplementary Figure S1**). Although the *k*-mer distribution was mathematically best fitted by a diploid model (*p* = 2), it lacked the characteristic two-peak pattern typically expected for a conventional diploid genome. Instead, a single dominant *k*-mer peak was observed at approximately 77× coverage, while the model estimated the genome composition as 73.3% homozygous (aa) and 26.7% heterozygous (ab). Despite the single-peak profile in the *k*-mer distribution, the substantial genomic heterogeneity indicates that SY16 is unlikely to be a typical haploid. Since *k-mer* based models alone are limited in resolving such structural complexities, further cytological validation is required.

The mitochondrial genome of *Pseudozyma* sp. SY16 was successfully assembled and annotated, revealing a circular genome of approximately 39,750 bp (**Supplementary Figure S2**). In addition to small and large mitochondrial rRNA subunits (rrnS, rrnL), the mitochondrial genome encodes ATP synthase complex components (atp6, 8 and 9), cytochrome B, cytochrome oxidase subunits (cox 1–3), NADH dehydrogenase subunits (nd1–6 and nd4l), small ribosomal subunit protein (rps 3), and 22 tRNA genes. Overall, the mitochondrial genome contains a comprehensive set of genes required for mitochondrial energy metabolism and protein synthesis, supporting the completeness and functional integrity of the assembled mitochondrial genome.

### 3.3 Phylogenetic placement and comparative genomics

Phylogenetic analysis based on the ITS gene sequence showed that *Pseudozyma* sp. SY16 clustered within a monophyletic clade containing *P. tsukubaensis* NBRC 1940 (**Supplementary Figure S3**). Whole-genome comparison between strain SY16 and *P. tsukubaensis* NBRC 1940 revealed an ANI value of 98.3% (**Figure 3**). These phylogenetic and genomic similarities support the identification of strain SY16 as *P. tsukubaensis*.

**Figure 3:**
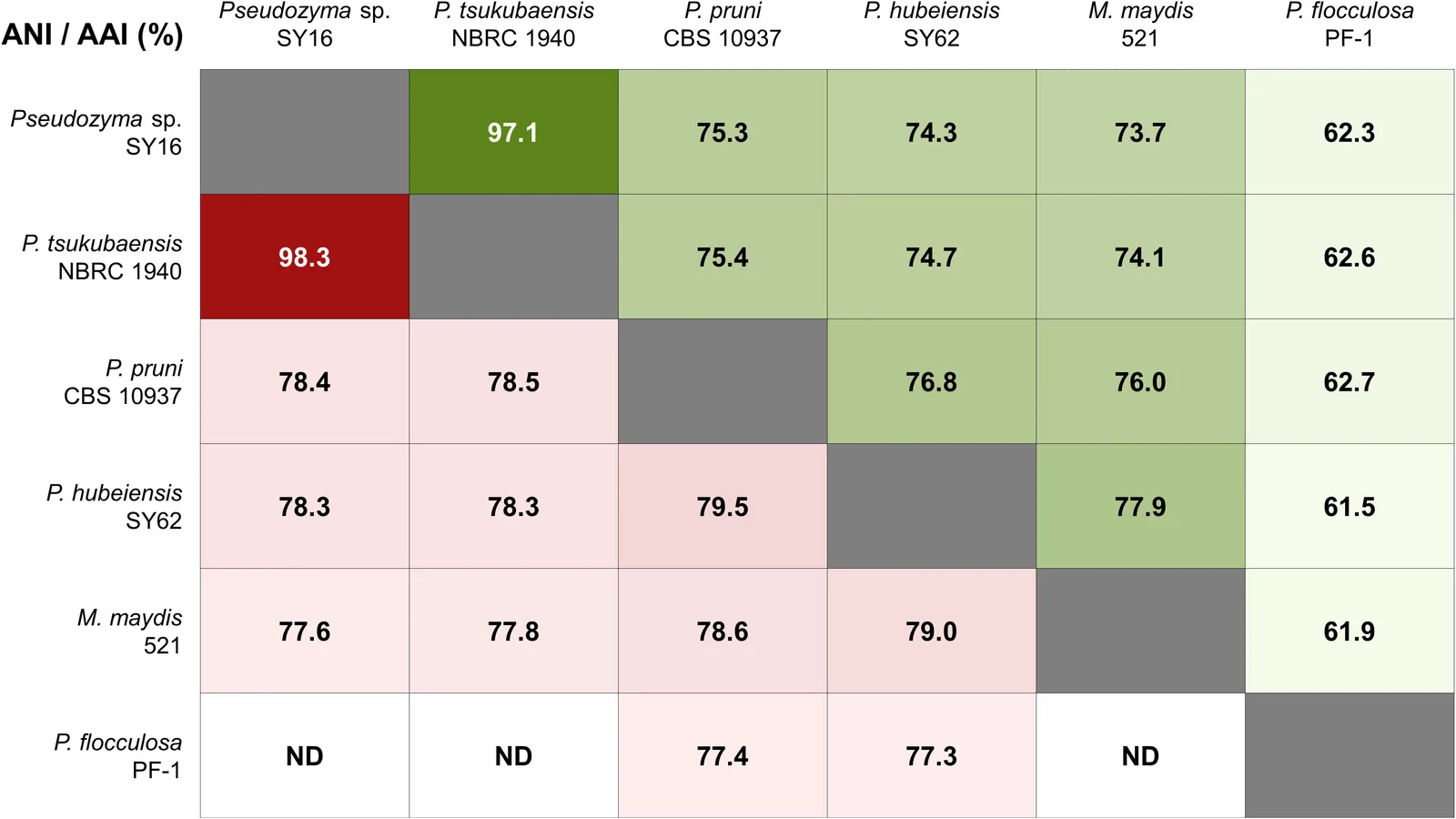
The average amino acid identity (AAI) and average nucleotide identity (ANI) values. In the comparative matrix, the upper right half displays AAI values, whereas the lower left half shows ANI values. ND, not determined - ANI could not be estimated by FastANI because insufficient homologous genomic regions were detected.

To assess genome-wide protein conservation, orthologous protein clustering was performed among *Pseudozyma* sp. SY16, *P. tsukubaensis* NBRC 1940, *P. pruni* CBS 10937, *P. hubeiensis* SY62, *M. maydis* 521, and *P. flocculosa* PF-1. The resulting phylogenetic tree is presented in **Figure 4A**, with *P. flocculosa* PF-1 positioned as the outgroup. The number of orthologous groups ranged from 5,442 in *P. flocculosa* PF-1 to 6,009 in *Pseudozyma* sp. SY16, with *P. tsukubaensis* NBRC 1940 (5,944), *P. pruni* CBS 10937 (5,776), *P. hubeiensis* SY62 (5,745), and *M. maydis* 521 (5,662) showing intermediate values (**Figure 4B**).

**Figure 4:**
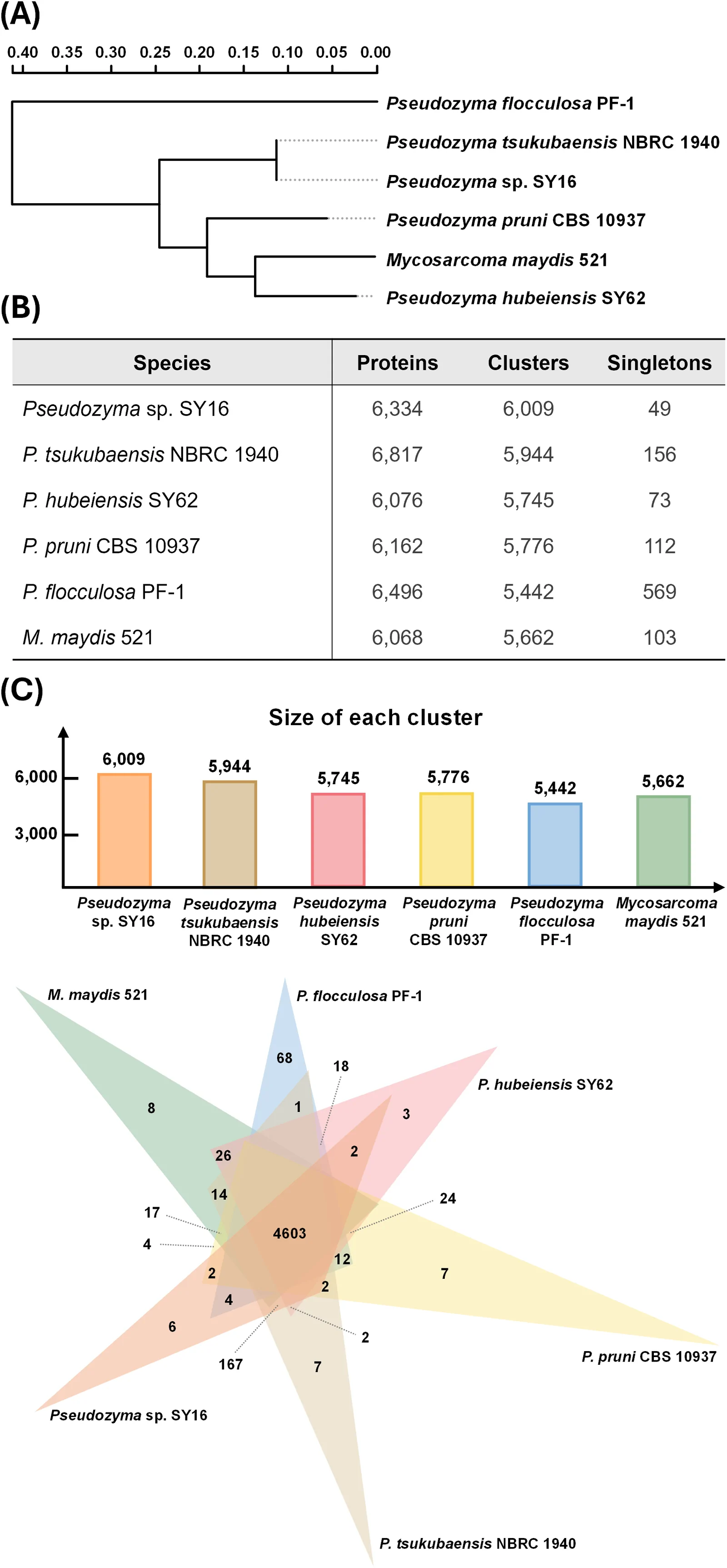
Pangenomic analysis. **(A)** phylogenetic tree based on single-copy orthologous genes, constructed using FastTree2 with the maximum-likelihood method with the JTT+CAT evolutionary model. **(B)** summary of the number of proteins, clusters, and single proteins within each genome. **(C)** Venn diagram showing the number of shared and unique protein clusters between SY16 and five related species.

Across the six genomes, 36,963 of 37,953 predicted genes were assigned to 6,397 orthogroups, corresponding to 97.4% of the total genes, while 990 genes (2.6%) remained unassigned. A total of 4,603 orthologous clusters were shared among all six species (**Figure 4C**). *Pseudozyma* sp. SY16 contained six species-specific clusters, all of which lacked informative GO annotations. In addition, 99 species-specific orthogroups comprising 328 genes were identified across the six species, representing 0.9% of the total genes, and 3,909 single-copy orthogroups were identified. Overall, these results demonstrate substantial genome-wide conservation among the examined species, while highlighting a small set of SY16-specific orthologous groups.

### 3.4 MEL biosynthetic gene cluster

The genomic organization of the MEL biosynthetic locus was examined across the six fungal species using clinker-based synteny analysis. The *mac1*, *mac2*, *mat1*, *mmf1*, and *emt1* genes of *Pseudozyma* sp. SY16 were used to compare the corresponding genomic regions in the related species. In *P. hubeiensis* SY62 and *M. maydis* 521, all five MEL biosynthetic genes were identified within syntenic genomic regions, and their relative positions and orientations were largely conserved with those in SY16 (**Figure 5**). The corresponding homologous genes showed sequence identities ranging from 48% to 70% in *P. hubeiensis* SY62 and from 52% to 69% in *M. maydis* 521, indicating substantial conservation of both gene content and genomic organization in these species.

**Figure 5:**
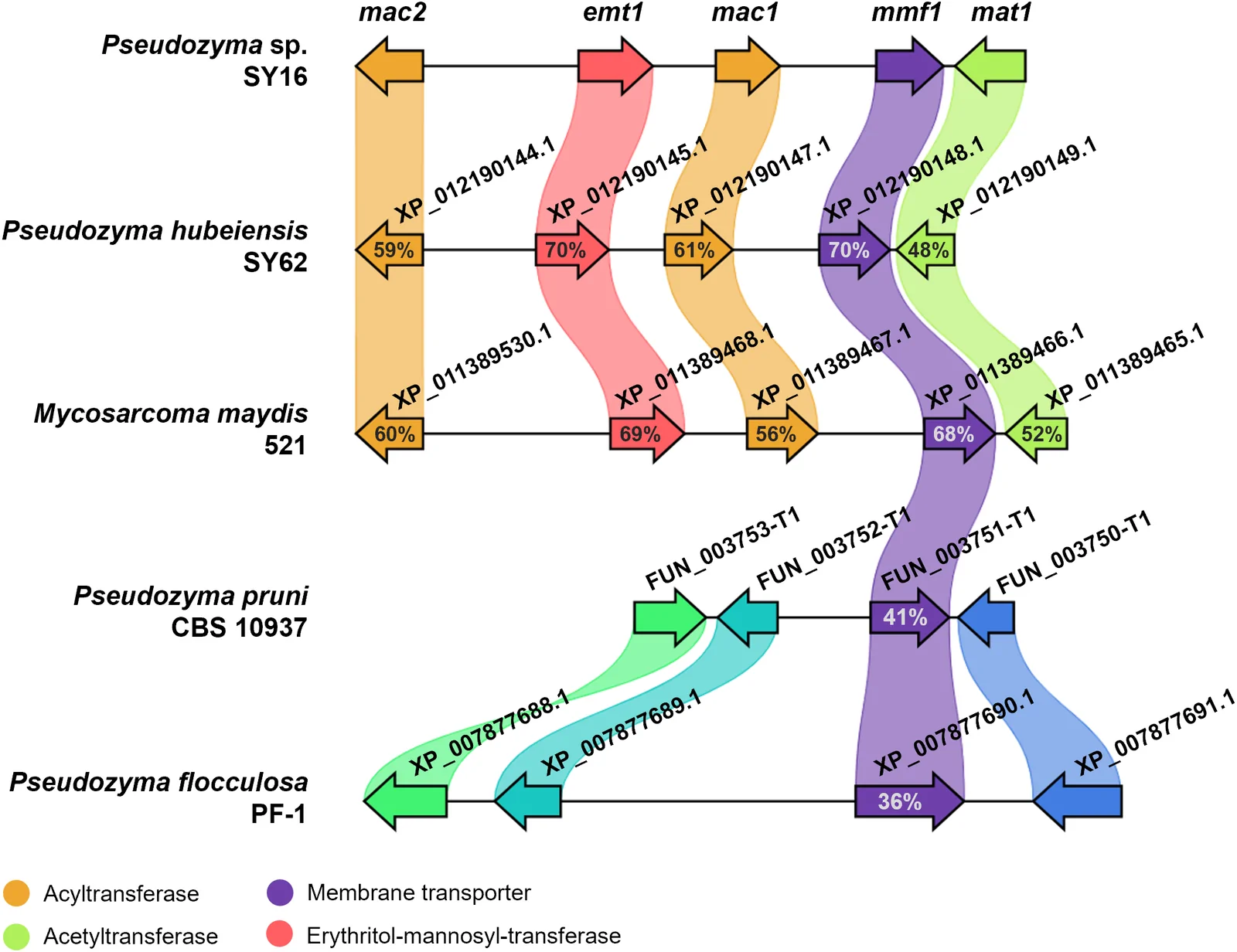
MEL biosynthesis. Comparison of the MEL biosynthetic gene cluster (BGC) of SY16 with those of related species. Homologous genes are connected by bands of the same color, and sequence identity values between SY16 MEL genes and their homologs are indicated. Accession numbers of the corresponding genes are provided. For *P. pruni*, Funannotate-derived protein identifiers are shown because the protein sequences were predicted.

In contrast, the MEL biosynthetic locus showed a markedly different pattern in *P. pruni* CBS 10937 and *P. flocculosa* PF-1. Among the five genes examined, only *mmf1* was identified in the corresponding syntenic regions, whereas the other MEL biosynthetic genes were not detected (**Figure 5**). The *mmf1* homologs showed lower sequence identities of 36–41% compared with the corresponding protein in SY16. Despite the lower sequence similarity, the conserved syntenic position of *mmf1* suggests that this gene has been retained in these species, whereas the remaining MEL biosynthetic genes appear to have undergone greater divergence or loss. Overall, the synteny analysis revealed a conserved MEL biosynthetic gene organization in *P. hubeiensis* and *M. maydis*, whereas substantial divergence in MEL-associated gene content was observed in *P. pruni* and *P. flocculosa*.

### 3.5 Genome annotation and characterization of mating-type

Of the 6,334 predicted protein-coding genes, 6,323 had complete coding sequences with both start and stop codons. One gene lacked both start and stop codons, while the remaining 10 genes had incomplete terminal regions, with six lacking a stop codon and four lacking a start codon.

Furthermore, to evaluate the carbohydrate metabolic potential of the genome, CAZymes were identified in the genome of *Pseudozyma* sp. SY16 by searching seven different classes: auxiliary activities (AA), proteins containing a carbohydrate-binding module (CBM), carbohydrate esterases (CE), glycoside hydrolases (GH), glycosyltransferases (GT), polysaccharide lyases (PL) and expansins (EXP). In *Pseudozyma* sp. SY16, a total of 115 proteins encoded for GH, 67 proteins encoded for GT, 27 proteins encoded for AA, 14 proteins encoded for CE, 4 proteins encoded for CBM, 2 proteins encoded for PL, and no protein for EXP. Among the identified CAZymes, GHs represented the largest proportion. Within the GHs, GH18, which is mainly involved in chitin degradation, was the most abundant family, accounting for 8.8% of the total CAZymes. The second most abundant family was GT2 associated with carbohydrate and cell wall polysaccharide biosynthesis, which accounted for 8.0% of the total CAZymes (**Supplementary Figure S5**).

In addition to primary carbohydrate metabolism, the genomic potential of *Pseudozyma* sp. SY16 for secondary metabolite biosynthesis was further investigated. According to the antiSMASH, a total of 12 putative secondary metabolite biosynthetic gene clusters (BGCs) were identified in the genome of *Pseudozyma* sp. SY16. This reveals diverse biosynthetic capacities that may contribute to the production of structurally and functionally varied secondary metabolites. Notably, two regions showed high homology to previously characterized biosynthetic pathways – a terpene cluster with 97% similarity to the previously characterized clavaric acid gene cluster, suggesting that this region may be involved in the biosynthesis of structurally related terpenoid compounds (**Supplementary Figure S6**). The second region was identified as a non-ribosomal peptide synthetase-like (NRPS-like) cluster, which showed 100% similarity to a previously reported choline-associated locus. The high similarity of these two regions indicates that *Pseudozyma* sp. SY16 may retain conserved biosynthetic pathways with potential roles in secondary metabolite production. However, further experimental validation, such as LC-MS-based metabolomic analysis, is required to identify the specific metabolites produced by these clusters and to characterize their biological functions.

BLASTP analysis identified homologs of the mating-type genes *bE1* and *bW1* in the SY16 genome (**Supplementary Table S2**). Both *bE1* and *bW1* showed high sequence similarity to previously reported mating-type genes, supporting their identification in the SY16 genome. The presence of both *bE1* and *bW1* suggests that SY16 contains the core genetic components of the *b* mating-type locus, which is associated with mating-type regulation in basidiomycete fungi. Overall, genome annotation revealed that *Pseudozyma* sp. SY16 possesses a diverse genetic repertoire associated with carbohydrate metabolism, secondary metabolite biosynthesis, and mating-type regulation, providing a genomic basis for further investigation of its metabolic and reproductive characteristics.

### 3.6 Nuclear organization revealed by confocal microscopy

To further investigate the unusual genomic pattern observed in the SY16 genome, the nuclear organization of SY16 cells was examined using DAPI staining and confocal microscopy. Cells exhibiting two distinct DAPI-stained nuclear signals were frequently observed, with approximately 50% of the cells examined showing a binucleate-like morphology (**Supplementary Figure S4**). Notably, cells undergoing division were also observed to contain two distinct nuclear signals, including cells in which the two nuclei appeared to be undergoing division (**Figure 6**). These observations indicated that two nuclei may frequently coexist within individual SY16 cells and prompted further investigation of their potential genetic relationship.

**Figure 6:**
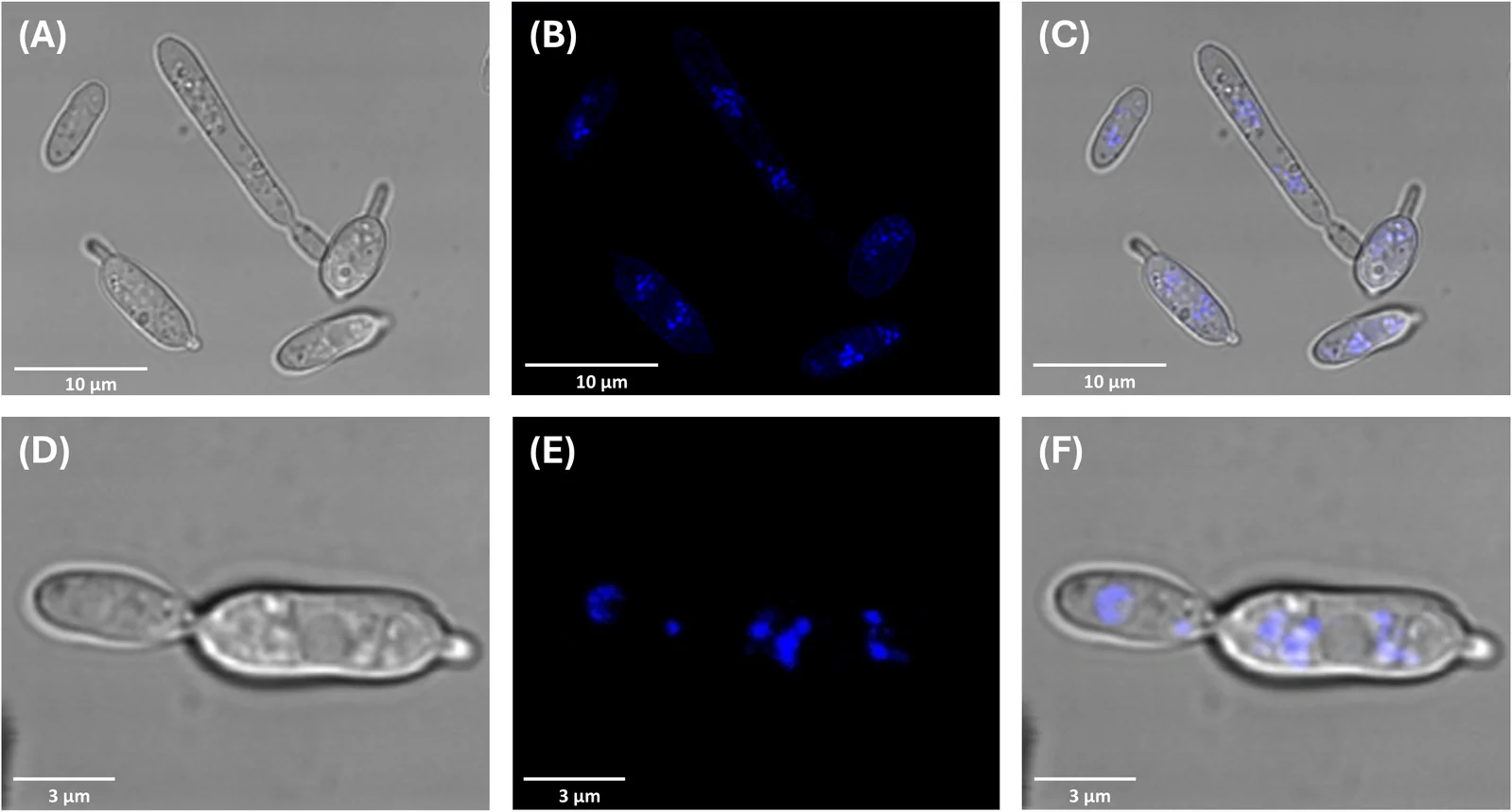
Confocal microscopic observations of *Pseudozyma* sp. SY16 cells stained with DAPI. **(A–C)** Micrographs of a representative cell showing **(A)** differential interference contrast (DIC), **(B)** DAPI-stained fluorescence confocal imaging, and **(C)** the merged image of DIC and DAPI fluorescence. Scale bar, 10 μm. **(D–F)** Micrographs of another representative cell displaying **(D)** DIC, **(E)** DAPI-stained fluorescence confocal imaging, and **(F)** the merged image of DIC and DAPI fluorescence. Scale bar, 3 μm.

To assess whether the observed binucleate morphology was associated with a dikaryotic state, the mating-type locus was examined in the SY16 genome. The *b* mating-type locus contained the *bE1* and *bW1* genes, whereas no additional *bE* or *bW* alleles were identified (**Supplementary Table S2**). The presence of a single *bE*/*bW* allele combination does not provide evidence for the coexistence of two genetically distinct mating-type nuclei within the same cell. Thus, although the frequent occurrence of binucleate cells is consistent with the presence of multiple nuclei, the current mating-type composition does not support a conventional dikaryotic state involving two distinct mating-type alleles.

Nevertheless, the combination of a substantial heterozygous component inferred from GenomeScope analysis and the frequent observation of binucleate cells suggests that the nuclear organization of SY16 may be more complex than that of a conventional haploid or diploid yeast. The two nuclei observed in the present mating-type analysis likely represent transient binucleate states associated with the cell cycle. Further analyses, including nucleus-specific genomic analysis or allele-specific sequencing, will be required to determine the genetic relationship between the two nuclei and to clarify the nuclear organization and ploidy of SY16.

## 4 Discussion

In this study, we generated a high-quality draft genome of *Pseudozyma* sp. SY16 and characterized its genomic, metabolic, and cellular features. The hybrid genome assembly consisted of 20.2 Mbp distributed across 23 nuclear scaffolds and one mitochondrial scaffold, with 98.9% of conserved Basidiomycota BUSCO genes recovered as complete. Compared with the highly fragmented genome of *P. tsukubaensis* NBRC 1940, which consists of 12,840 scaffolds, the markedly improved continuity of the SY16 assembly provides a reliable genomic framework for comparative genomics and functional studies. This resource is particularly valuable given the high MEL production capacity of SY16 and its potential as a microbial production host. ITS-based phylogenetic analysis and a whole-genome ANI value of 98.3% further support the assignment of SY16 to *P. tsukubaensis*, while the improved assembly provides a strain-specific genomic reference for investigating the genetic basis of its distinctive phenotypic and metabolic properties. While recent taxonomic revisions place several *Pseudozyma* species into *Moesziomyces* or related genera, we tentatively refer to strain SY16 as *Pseudozyma* sp. SY16 (affiliated with *P. tsukubaensis*) in accordance with historical literature on MEL production.

Genome-wide orthologous clustering revealed a high degree of conservation among the examined *Pseudozyma* and related species, with 97.4% of predicted genes assigned to orthologous groups and 4,603 orthologous clusters shared across all six genomes. This extensive conservation suggests that these species retain a common genomic backbone despite their differences in ecological characteristics and metabolic capabilities. However, the evolutionary pattern of the MEL biosynthetic locus did not completely mirror the genome-wide phylogenetic relationship. In the ortholog-based phylogenetic tree, *P. flocculosa* PF-1 was positioned as the most divergent species, whereas *P. pruni* CBS 10937 was placed relatively close to SY16. Nevertheless, the complete set of MEL-associated genes examined in this study was conserved in SY16, *P. hubeiensis* SY62, and *M. maydis* 521, whereas only *mmf1* was detected in the corresponding syntenic regions of *P. pruni* and *P. flocculosa*. Thus, conservation of the MEL biosynthetic locus does not appear to strictly follow genome-wide phylogenetic relationships. This discordance suggests that the evolutionary history of the MEL biosynthetic locus may have been shaped by lineage-specific processes, including differential gene retention, gene loss, sequence divergence, or genomic rearrangement, rather than solely by overall species divergence. Additional genomes representing both MEL-producing and non-MEL-producing lineages will be necessary to determine whether the observed distribution reflects repeated loss of MEL biosynthetic genes or differential retention of an ancestral biosynthetic locus.

The conservation of the MEL biosynthetic genes in SY16, *P. hubeiensis*, and *M. maydis* is particularly notable because both gene content and genomic organization were largely maintained in these species. The preservation of *mac1*, *mac2*, *mat1*, *mmf1*, and *emt1* within syntenic regions suggests that these genes may have been maintained as a functionally associated biosynthetic module. In contrast, the retention of *mmf1* in *P. pruni* and *P. flocculosa* despite the absence of the other examined MEL-associated genes raises the possibility that individual components of the ancestral locus have experienced different evolutionary constraints. The conserved syntenic position of *mmf1* suggests that this gene may have been subject to distinct evolutionary constraints, although its functional significance in these species remains to be determined. Notably, while the MEL production capacity of *P. pruni* has not yet been experimentally evaluated, previous studies have clearly established that *P. flocculosa* fails to produce MELs (Morita et al., 2007b). This phenotypic absence of MEL production in *P. flocculosa* is remarkably consistent with our genomic observation.

These differences in the conservation of MEL biosynthetic genes may also be related to the ecological functions of MEL production. MELs are extracellular glycolipids with amphiphilic properties and have been proposed to contribute to interactions between yeast cells and their surrounding environments, including surface-associated growth and the utilization of hydrophobic substrates (Ron and Rosenberg, 2001; Arutchelvi et al., 2008). Such functions may provide a fitness advantage under particular environmental conditions, potentially favoring the retention of the MEL biosynthetic pathway in some lineages. Conversely, when these functions provide limited benefit, the selective pressure to maintain the entire pathway may be reduced, allowing individual genes or parts of the locus to diverge or be lost. The contrasting patterns observed among the examined species are consistent with this possibility, although the ecological factors underlying the differential retention of MEL biosynthetic genes remain unclear.

Importantly, the presence of a complete MEL biosynthetic gene set alone is unlikely to fully explain the high MEL productivity of SY16. Biosynthetic output is likely influenced not only by the structural genes themselves but also by transcriptional regulation, precursor availability, fatty-acid metabolism, lipid transport, and cellular responses to the hydrophobic substrates used for MEL production. Thus, the conserved MEL biosynthetic locus in SY16 may provide the genetic capacity for MEL production, whereas its high production phenotype may result from additional regulatory and metabolic features that remain to be characterized.

Beyond MEL biosynthesis, genome annotation revealed a broad metabolic repertoire in SY16, including diverse carbohydrate-active enzymes and 12 putative secondary metabolite biosynthetic gene clusters. The high representation of GH18 and GT2 further highlights the metabolic versatility encoded in the SY16 genome. The abundance of GH18 enzymes may facilitate the breakdown of chitin and other structurally complex carbohydrates, whereas GT2 enzymes are involved in the biosynthesis of carbohydrate-containing cellular components. Together, these findings indicate that SY16 has genomic potential for both the degradation of environmental carbohydrates and the synthesis of cell wall-associated polysaccharides, which may contribute to its ability to adapt to diverse nutritional and environmental conditions. Consistent with this broad metabolic capacity, antiSMASH analysis identified additional secondary metabolite biosynthetic regions, including terpene and NRPS-like clusters, suggesting that the biosynthetic potential of SY16 extends beyond MEL production. Although the products and biological functions of these predicted clusters remain to be experimentally validated, their presence indicates that SY16 may possess unexplored capacity for the production of diverse secondary metabolites. Thus, the high-quality genome generated in this study provides an important foundation for prioritizing candidate biosynthetic pathways and exploring metabolic engineering strategies beyond the currently characterized MEL pathway.

An additional feature distinguishing SY16 from a conventional yeast model was its unusual nuclear organization. GenomeScope analysis revealed a substantial heterozygous component, although the *k*-mer distribution lacked the characteristic two-peak pattern expected for a conventional diploid genome. This estimated heterogeneity (26.7%) from the short-read *k*-mer model could reflect either substantial sequence divergence between duplicated chromosomes/nuclei, or technical artifacts arising from repetitive regions and segmental duplications. Nonetheless, the absence of a distinct second heterozygous *k*-mer peak highlights the non-canonical nature of its genome structure. At the same time, DAPI staining frequently revealed two distinct nuclear signals within individual cells. The coexistence of these genomic and cytological observations suggests that SY16 possesses a complex nuclear organization that cannot be readily explained by a simple haploid or diploid model. Importantly, the presence of two nuclei does not by itself establish a conventional dikaryotic state. The identification of a single *bE1*/*bW1* allele combination without evidence for additional distinct *bE* or *bW* alleles argues against a typical dikaryon composed of two genetically distinct mating-type nuclei. Instead, SY16 may undergo a multinucleate or transient binucleate state in which nuclear number and genetic composition are not necessarily equivalent to the canonical dikaryotic organization observed in sexually differentiated basidiomycetes.

The nuclear phenotype is particularly interesting in relation to the morphological development of SY16. The strain underwent a stable transition from yeast-like growth to pseudohyphal morphology during cultivation on solid medium, and the pseudohyphal phenotype was maintained following subculturing. In basidiomycetous fungi, filamentous growth and mating are frequently interconnected with developmental signaling pathways and changes in nuclear organization (Boyce and Andrianopoulos, 2015; Kijpornyongpan and Aime, 2020). However, the biological significance of the binucleate state observed in SY16 remains unclear. The coexistence of pseudohyphal morphology and binucleate cells may indicate an association between morphological development and nuclear organization in this strain, although the present observations do not establish a direct relationship between these features. Further studies will be required to determine whether binucleate cells represent a stable characteristic of SY16 or occur preferentially during specific stages of growth or morphological development.

The unusual nuclear organization may also have practical implications for genetic engineering of SY16. In our preliminary gene disruption trials using a hygromycin resistance gene as a selectable marker, transformants frequently harbored both wild-type and disrupted sequences, suggesting that selection of resistant colonies does not necessarily correspond to genetic homogenization of the entire cellular population. In a binucleate background, genome editing could potentially occur in only a subset of nuclei or genomic copies, which may explain the coexistence of wild-type and edited sequences within the same colony or cell lineage. Such a cellular organization could complicate conventional genotype validation and may contribute to difficulties in obtaining genetically uniform knockout strains.

Taken together, the present study demonstrates that SY16 is not only a high-producing MEL yeast with a well-conserved biosynthetic locus but also a genetically and cellularly distinctive *Pseudozyma* strain. The discordant evolutionary patterns between the genome-wide phylogeny and MEL biosynthetic locus suggest that specialized metabolic pathways may undergo lineage-specific evolutionary trajectories that are not necessarily reflected by overall species relationships. At the same time, the combination of substantial genomic heterogeneity, frequent binucleate cells, and stable morphological differentiation points to an unusual relationship between genome organization and cellular development. These findings broaden our understanding of the biological characteristics of SY16 and provide a genomic basis for improving its use as a non-model microbial production host. In particular, resolving the relationship between nuclear organization, genome heterogeneity, and genetic transformation will be an important step toward establishing more predictable genome engineering strategies and exploiting the metabolic potential of SY16 for MEL and other value-added bioproducts.

## Data Availability

The full DNA-sequencing and metadata associated with this study have been deposited in NCBI GenBank under the following accession numbers: BioProject PRJNA1498694, Biosample SAMN61854671 and WGS nuclear genome JCARYC000000000.

## References

[B1] Adu S. A. Twigg M. S. Naughton P. J. Marchant R. Banat I. M. (2023). Characterisation of cytotoxicity and immunomodulatory effects of glycolipid biosurfactants on human keratinocytes. Appl. Microbiol. Biotechnol. 107, 137–152. doi: 10.1007/s00253-022-12302-5 36441210 PMC9750927

[B2] Arutchelvi J. I. Bhaduri S. Uppara P. V. Doble M. (2008). Mannosylerythritol lipids: a review. J. Ind. Microbiol. Biotechnol. 35, 1559–1570. doi: 10.1007/s10295-008-0460-4 18716809

[B3] Banat I. M. Franzetti A. Gandolfi I. Bestetti G. Martinotti M. G. Fracchia L. . (2010). Microbial biosurfactants production, applications and future potential. Appl. Microbiol. Biotechnol. 87, 427–444. doi: 10.1007/s00253-010-2589-0 20424836

[B4] Blin K. Shaw S. Vader L. Szenei J. Reitz Z. L. Augustijn H. E. . (2025). antiSMASH 8.0: extended gene cluster detection capabilities and analyses of chemistry, enzymology, and regulation. Nucleic Acids Res. 53, W32–W38. doi: 10.1093/nar/gkaf334 40276974 PMC12230676

[B5] Boyce K. J. Andrianopoulos A. (2015). Fungal dimorphism: the switch from hyphae to yeast is a specialized morphogenetic adaptation allowing colonization of a host. FEMS Microbiol. Rev. 39, 797–811. doi: 10.1093/femsre/fuv035 26253139

[B6] Emms D. M. Kelly S. (2019). OrthoFinder: phylogenetic orthology inference for comparative genomics. Genome Biol. 20, 238. doi: 10.1186/s13059-019-1832-y 31727128 PMC6857279

[B7] Fei D. Zhou G. W. Yu Z. Q. Gang H. Z. Liu J. F. Yang S. Z. . (2020). Low‐toxic and nonirritant biosurfactant surfactin and its performances in detergent formulations. J. Surfactants Detergents 23, 109–118. doi: 10.1002/jsde.1235642587375

[B8] Fernandes N. A. T. Simões L. A. Dias D. R. (2023). Comparison of biodegradability, and toxicity effect of biosurfactants with synthetic surfactants. Adv. Biosurfactants Res., 117–136. doi: 10.1007/978-3-031-21682-4_625040404

[B9] Fukuoka T. Morita T. Konishi M. Imura T. Sakai H. Kitamoto D. (2007). Structural characterization and surface-active properties of a new glycolipid biosurfactant, mono-acylated mannosylerythritol lipid, produced from glucose by *Pseudozyma antarctica*. Appl. Microbiol. Biotechnol. 76, 801–810. doi: 10.1007/s00253-007-1051-4 17607573

[B10] Gilchrist C. L. Chooi Y. H. (2021). Clinker & clustermap. js: automatic generation of gene cluster comparison figures. Bioinform. 37, 2473–2475. doi: 10.1093/bioinformatics/btab007 33459763

[B11] Greiner S. Lehwark P. Bock R. (2019). OrganellarGenomeDRAW (OGDRAW) version 1.3.1: expanded toolkit for the graphical visualization of organellar genomes. Nucleic Acids Res. 47, W59–W64. doi: 10.1093/nar/gkz238 30949694 PMC6602502

[B12] Han J. An Y. (2019). Characterization of the complete mitochondrial genome of *Pseudozyma* sp. JCC207. Mitochondrial DNA. Part. B. Resour. 4, 2084–2085. doi: 10.1080/23802359.2019.161662837339054

[B13] Hewald S. Linne U. Scherer M. Marahiel M. A. Kämper J. Bölker M. (2006). Identification of a gene cluster for biosynthesis of mannosylerythritol lipids in the basidiomycetous fungus *Ustilago maydis*. Appl. Environ. Microbiol. 72, 5469–5477. doi: 10.1128/AEM.00506-06 16885300 PMC1538720

[B14] Jimoh A. A. Lin J. (2019). Biosurfactant: A new frontier for greener technology and environmental sustainability. Ecotoxicol. Environ. Saf. 184, 109607. doi: 10.1016/j.ecoenv.2019.109607 31505408

[B15] Karnwal A. Shrivastava S. Al-Tawaha A. R. M. S. Kumar G. Singh R. Kumar A. . (2023). Microbial biosurfactant as an alternate to chemical surfactants for application in cosmetics industries in personal and skin care products: a critical review. BioMed. Res. Int. 2023, 2375223. doi: 10.1155/2023/2375223 37090190 PMC10118887

[B16] Katoh K. Rozewicki J. Yamada K. D. (2019). MAFFT online service: multiple sequence alignment, interactive sequence choice and visualization. Brief. Bioinform. 20, 1160–1166. doi: 10.1093/bib/bbx108 28968734 PMC6781576

[B17] Kijpornyongpan T. Aime M. C. (2020). Investigating the smuts: Common cues, signaling pathways, and the role of *MAT* in dimorphic switching and pathogenesis. J. Fungi 6, 368. doi: 10.3390/jof6040368 33339287 PMC7766764

[B18] Kim H. S. Jeon J. W. Kim B. H. Ahn C. Y. Oh H. M. Yoon B. D. (2006). Extracellular production of a glycolipid biosurfactant, mannosylerythritol lipid, by *Candida* sp. SY16 using fed-batch fermentation. Appl. Microbiol. Biotechnol. 70, 391–396. doi: 10.1007/s00253-005-0092-9 16133323

[B19] Kim H. S. Jeon J. W. Kim S. B. Oh H. M. Kwon T. J. Yoon B. D. (2002b). Surface and physico-chemical properties of a glycolipid biosurfactant, mannosylerythritol lipid, from *Candida antarctica*. Biotechnol. Lett. 24, 1637–1641. doi: 10.1023/A:102030981654541886696

[B20] Kim H. S. Jeon J. W. Lee H. W. Park Y. I. Seo W. T. Oh H. M. . (2002a). Extracellular production of a glycolipid biosurfactant, mannosylerythritol lipid, from *Candida antarctica*. Biotechnol. Lett. 24, 225–229. doi: 10.1023/A:101410042285741886696

[B21] Kim H. S. Lee C. H. Suh H. H. Ahn K. H. Oh H. M. Kwon G. S. . (1997). A lipopeptide biosurfactant produced by Bacillus subtilis C9 selected through the oil film-collapsing assay. J. Microbiol. Biotechnol. 7, 180–188.

[B22] Kim H. S. Yoon B. D. Choung D. H. Oh H. M. Katsuragi T. Tani Y. (1999). Characterization of a biosurfactant, mannosylerythritol lipid produced from *Candida* sp. SY16. Appl. Microbiol. Biotechnol. 52, 713–721. doi: 10.1007/s002530051583 10570818

[B23] Kunitake E. Tanaka T. Ueda H. Endo A. Yarimizu T. Katoh E. . (2019). CRISPR/Cas9-mediated gene replacement in the basidiomycetous yeast *Pseudozyma antarctica*. Fungal Genet. Biol. 130, 82–90. doi: 10.1016/j.fgb.2019.04.012 31026589

[B24] Marçais G. Kingsford C. (2011). A fast, lock-free approach for efficient parallel counting of occurrences of *k*-mers. Bioinform. 27, 764–770. doi: 10.1093/bioinformatics/btr011 21217122 PMC3051319

[B25] Morita T. Konishi M. Fukuoka T. Imura T. Kitamoto D. (2007a). Physiological differences in the formation of the glycolipid biosurfactants, mannosylerythritol lipids, between *Pseudozyma antarctica* and *Pseudozyma aphidis*. Appl. Microbiol. Biotechnol. 74, 307–315. doi: 10.1007/s00253-006-0672-3 17103161

[B26] Morita T. Konishi M. Fukuoka T. Imura T. Kitamoto H. K. Kitamoto D. (2007b). Characterization of the genus *Pseudozyma* by the formation of glycolipid biosurfactants, mannosylerythritol lipids. FEMS Yeast Res. 7, 286–292. doi: 10.1111/j.1567-1364.2006.00154.x 17328742

[B27] Morita T. Takashima M. Fukuoka T. Konishi M. Imura T. Kitamoto D. (2010). Isolation of basidiomycetous yeast *Pseudozyma tsukubaensis* and production of glycolipid biosurfactant, a diastereomer type of mannosylerythritol lipid-B. Appl. Microbiol. Biotechnol. 88, 679–688. doi: 10.1007/s00253-010-2762-5 20652239

[B28] Palmer J. M. Stajich J. (2020). Funannotate v1. 8.1: Eukaryotic genome annotation. Zenodo. doi: 10.5281/zenodo.113447742270437

[B29] Ranallo-Benavidez T. R. Jaron K. S. Schatz M. C. (2020). GenomeScope 2.0 and Smudgeplot for reference-free profiling of polyploid genomes. Nat. Commun. 11, 1432. doi: 10.1038/s41467-020-14998-3 32188846 PMC7080791

[B30] Ron E. Z. Rosenberg E. (2001). Natural roles of biosurfactants: Minireview. Environ. Microbiol. 3, 229–236. doi: 10.1046/j.1462-2920.2001.00190.x 11359508

[B31] Simão F. A. Waterhouse R. M. Ioannidis P. Kriventseva E. V. Zdobnov E. M. (2015). BUSCO: assessing genome assembly and annotation completeness with single-copy orthologs. Bioinform. 31, 3210–3212. doi: 10.1093/bioinformatics/btv351 26059717

[B32] Sun J. Lu F. Luo Y. Bie L. Xu L. Wang Y. (2023). OrthoVenn3: an integrated platform for exploring and visualizing orthologous data across genomes. Nucleic Acids Res. 51, W397–W403. doi: 10.1093/nar/gkad313 37114999 PMC10320085

[B33] Tran Q. G. Ryu A. J. Choi Y. J. Jeong K. J. Kim H. S. Lee Y. J. (2022). Enhanced production of biosurfactants through genetic engineering of *Pseudozyma* sp. SY16. Korean J. Chem. Eng. 39, 997–1003. doi: 10.1007/s11814-021-1020-030311153

[B34] Uliano-Silva M. Ferreira J. G. R. N. Krasheninnikova K. Consortium D. T. L. Formenti G. Abueg L. . (2023). MitoHiFi: a python pipeline for mitochondrial genome assembly from PacBio high fidelity reads. BMC Bioinform. 24, 288. doi: 10.1186/s12859-023-05385-y 37464285 PMC10354987

[B35] Van Den Belt M. Gilchrist C. Booth T. J. Chooi Y. H. Medema M. H. Alanjary M. (2023). CAGECAT: The CompArative GEne Cluster Analysis Toolbox for rapid search and visualisation of homologous gene clusters. BMC Bioinform. 24, 181. doi: 10.1186/s12859-023-05311-2 37131131 PMC10155394

[B36] Wong T. K. F. Ly-Trong N. Ren H. Demotte P. Banos H. Roger A. J. . (2026). IQ-TREE 3: phylogenomic inference software using complex evolutionary models. Mol. Biol. Evol. 43 (5), msag117. doi: 10.1093/molbev/msag117 42085559 PMC13191116

[B37] Zheng J. Ge Q. Yan Y. Zhang X. Huang L. Yin Y. (2023). dbCAN3: automated carbohydrate-active enzyme and substrate annotation. Nucleic Acids Res. 51, W115–W121. doi: 10.1093/nar/gkad328 37125649 PMC10320055

