# Supplementary material for: Draft genome sequence and phenotypic analysis of Pseudozyma sp. SY16 with evidence of binucleate cells
Source: Front Fungal Biol. 2026 Oct 29;7:2001516. doi: 10.3389/ffunb.2026.2001516 (PMC13755194; doi:10.3389/ffunb.2026.2001516)
Supplement: Supplementary file 1 [file DataSheet1.pdf]

Supplementary materials

Draft Genome Sequence and Phenotypic Analysis of *Pseudozyma* sp. SY16 with Evidence of Binucleate Cells

Ami Rho[1], Dong-Yun Choi[1], Jin-Ho Yun[1,2], Youngshin Park[1], Hong Il Choi[1,2], Dae-Hyun Cho[1], Hee-Sik Kim[1,2*], Yong Jae Lee[1,2*]

[1]Cell Factory Research Center, Korea Research Institute of Bioscience and Biotechnology, Daejeon 34141, South Korea

[2]Department of Environmental Biotechnology, University of Science and Technology, Daejeon 34113, South Korea

*** Correspondence:** Hee-Sik Kim and Yong Jae Lee (hkim@kribb.re.kr; leeyj@kribb.re.kr)

Keywords: Yeast, *Pseudozyma* sp. SY16, Mannosylerythritol lipids (MELs), *Pseudozyma*, Whole genome sequence, PacBio-Illumina

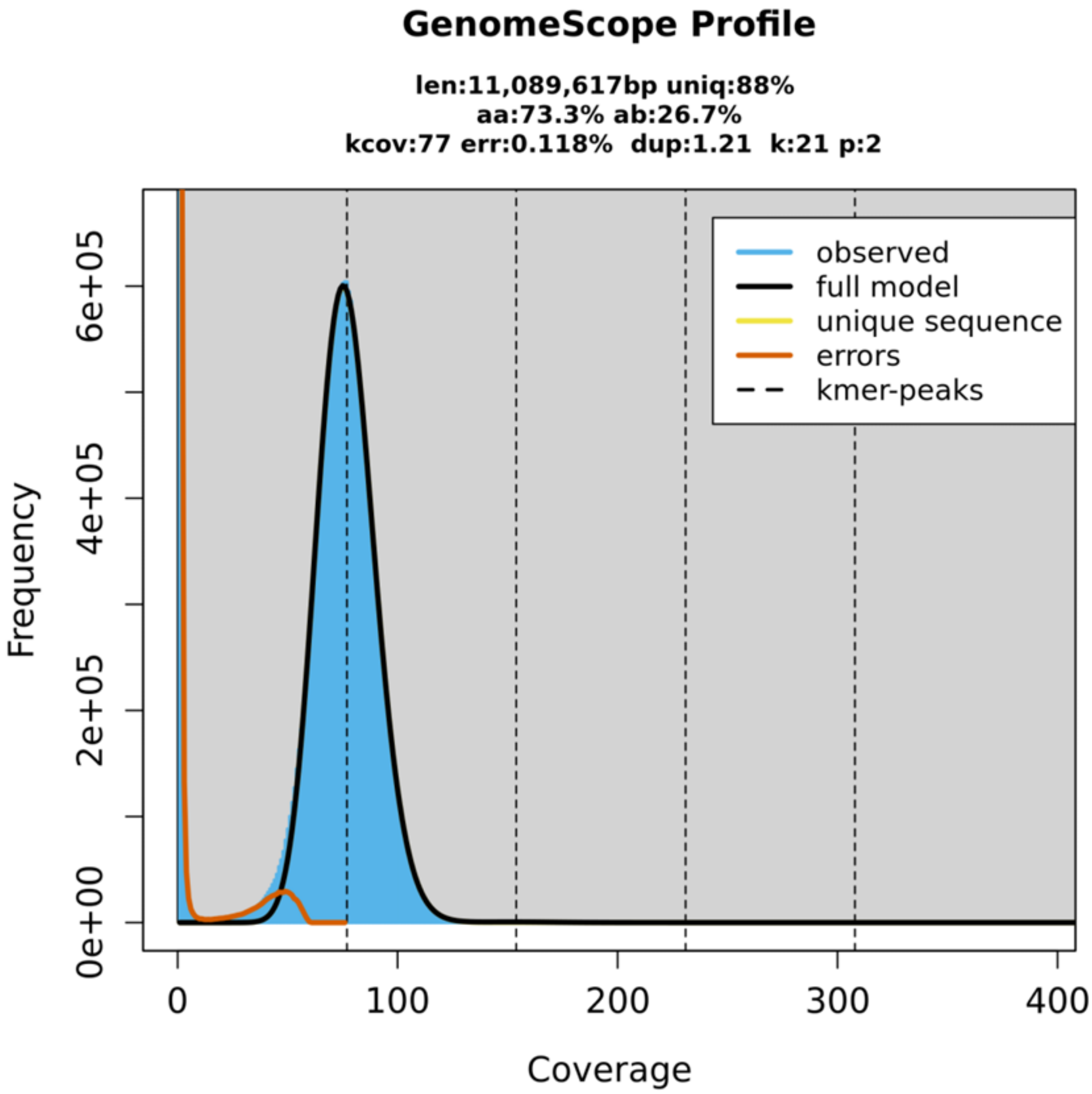

Figure S1. GenomeScope analysis of 21 *k*-mer profiles of *Pseudozyma* sp. SY16. Overall heterozygosity rate estimates are shown in each graph.

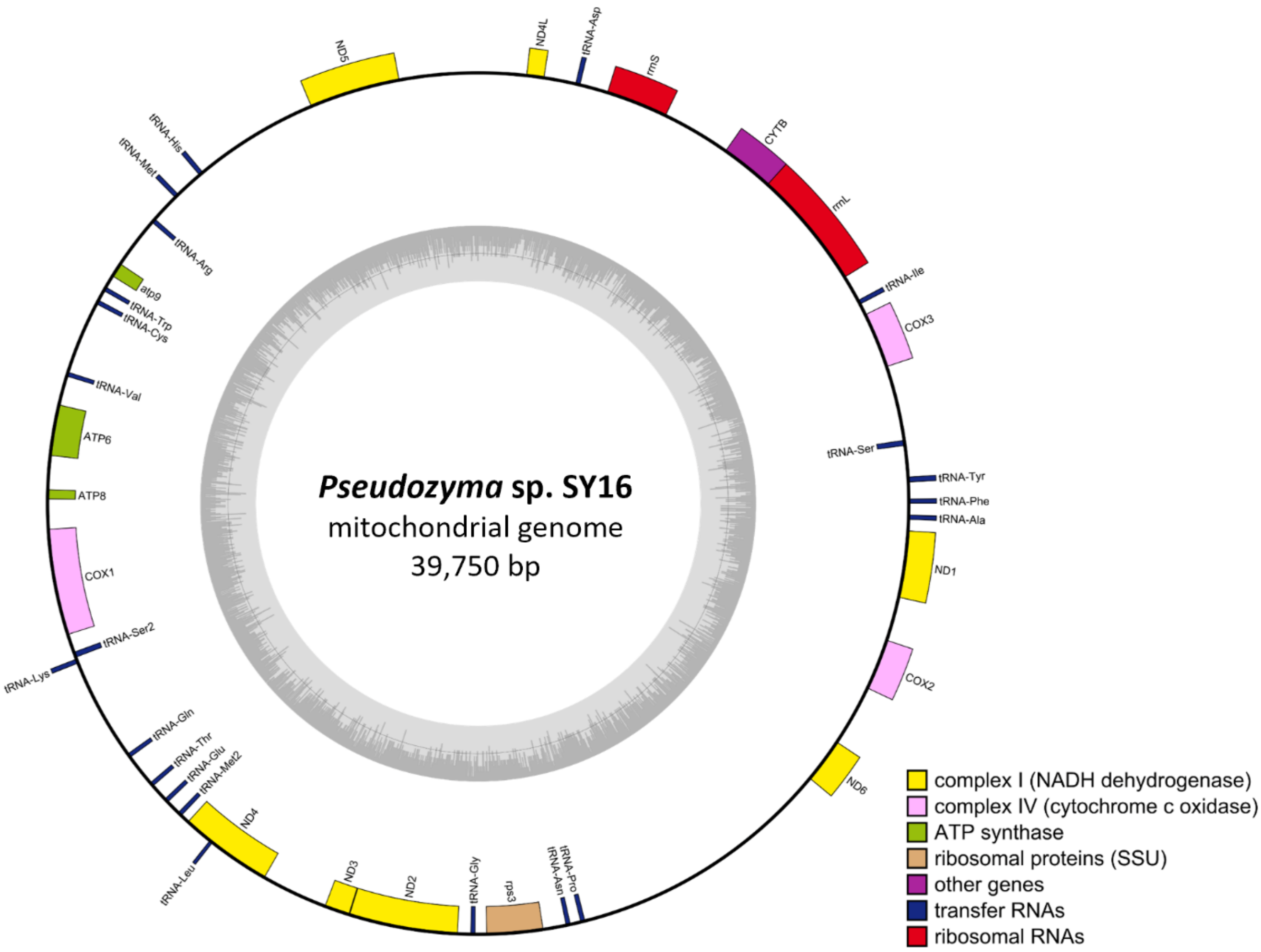

Figure S2. *Pseudozyma* sp. SY16 mitochondrial genome map.

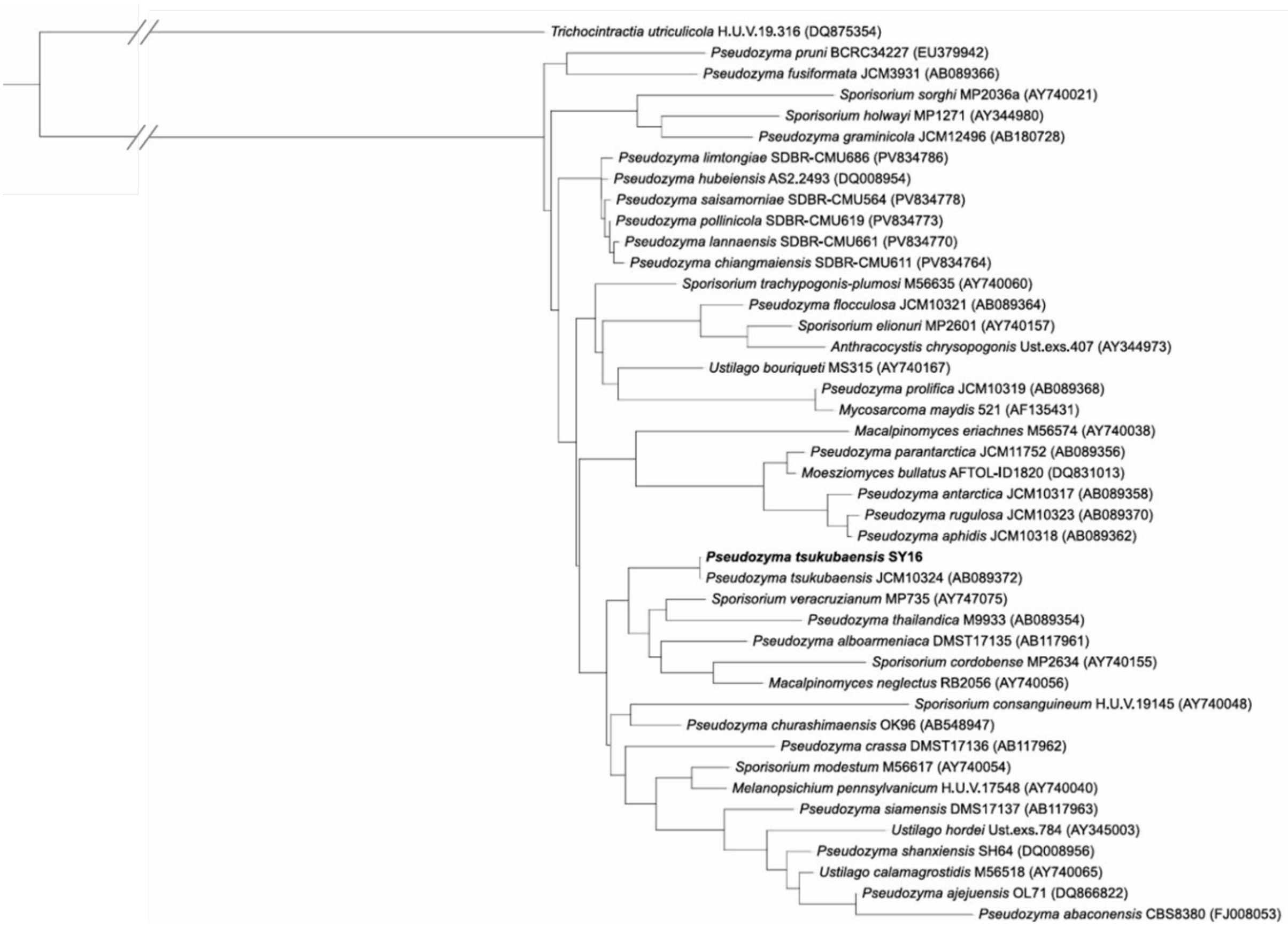

Figure S3. Phylogenetic tree of *Pseudozyma* sp. SY16 and related species based on ITS region sequences. The tree was constructed using the Maximum Likelihood method via IQ-TREE. The best-fit nucleotide substitution model was automatically selected using ModelFinder Plus. Numbers at the nodes indicate ultrafast bootstrap values based on 1,000 replicates. *Trichocintractia utriculicola* H.U.V. 19.316 was used as the outgroup.

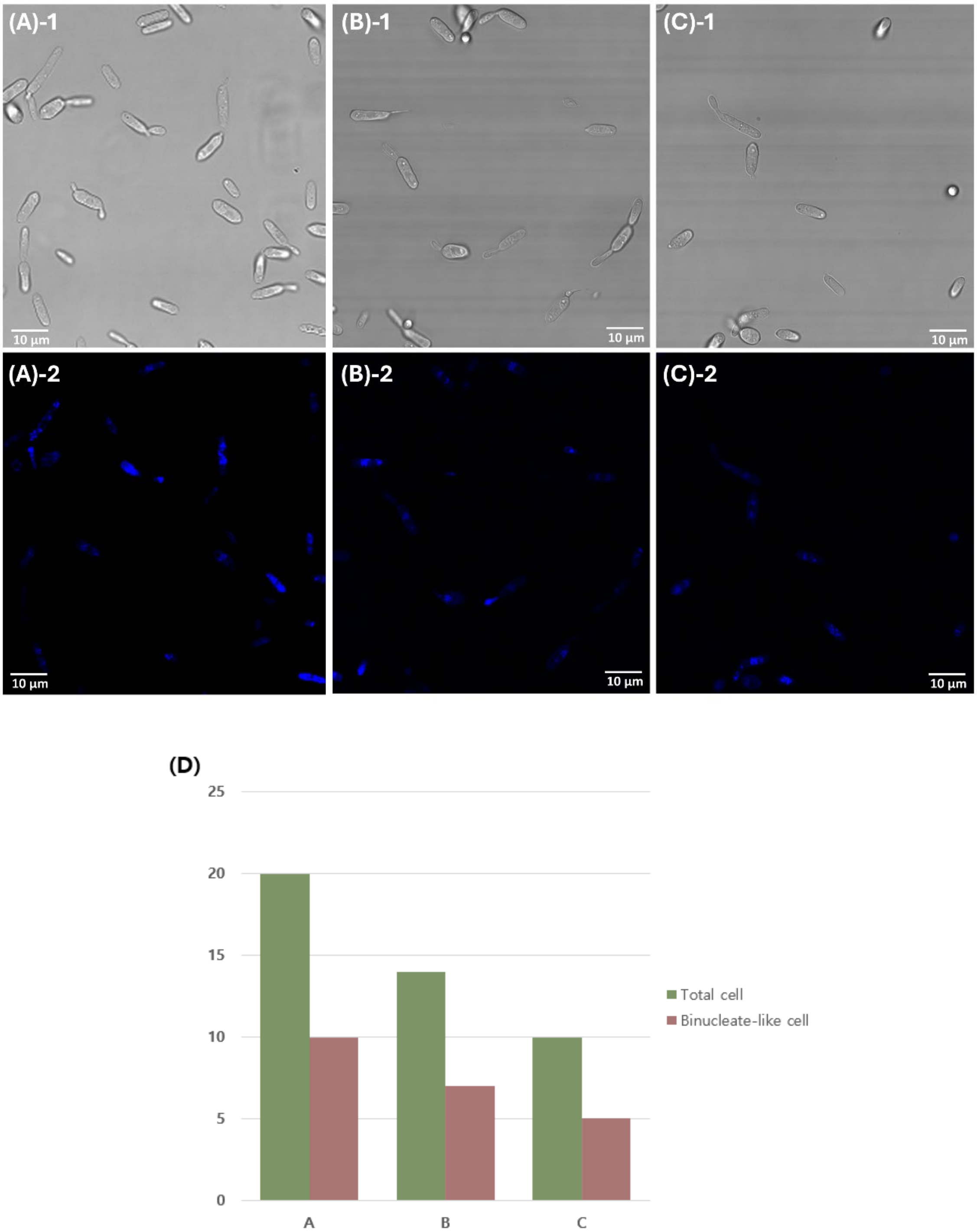

Figure S4. Cell morphology and quantification of binucleate cells. (A-C) Overview (zoomed-out) images of cells. (A-1, B-1, C-1) Differential interference contrast (DIC) micrographs displaying whole cell morphology. (A-2, B-2, C-2) Confocal fluorescence micrographs showing nuclear DNA stained with DAPI. (D) Quantification of the proportion of binucleate cells relative to the total number of cells per field of view. A, B, and C binucleate-like cells consistently accounted for approximately 50% of total cells. Data are presented independent field views. Scale bars represent 10 µm.

Table S1. Strains used for ITS phylogenetic analysis and their accession numbers.

Num.	Species	Strain	Accession Number
1	*Anthracocystis chrysopogonis*	Ust.exs.407	AY344973
2	*Anthracocystis destruens*	Ust.exe.472	AY344976
3	*Macalpinomyces eriachnes*	M 56574	AY740038
4	*Macalpinomyces neglectus*	RB2056	AY740056
5	*Melanopsichium pennsylvanicum*	HUV17548	AY740040
6	*Moesziomyces bullatus*	AFTOL-ID 1820	DQ831013
7	*Mycosarcoma maydis*	521	AF135431
8	*Pseudozyma abaconensis*	CBS8380	FJ008053
9	*Pseudozyma alboarmeniaca*	DMST17135	AB117961
10	*Pseudozyma antarctica*	JCM10317	AB089358
11	*Pseudozyma aphidis*	JCM10318	AB089362
12	*Pseudozyma chiangmaiensis*	SDBR-CMU611	PV834764
13	*Pseudozyma churashimaensis*	OK96	AB548947
14	*Pseudozyma crassa*	DMST17136	AB117962
15	*Pseudozyma flocculosa*	JCM10321	AB089364
16	*Pseudozyma fusiformata*	JCM3931	AB089366
17	*Pseudozyma graminicola*	JCM12496	AB180728
18	*Pseudozyma hubeiensis*	AS2.2493	DQ008954
19	*Pseudozyma jejuensis*	OL71	DQ866822
20	*Pseudozyma lannaensis*	SDBR-CMU661	PV834770
21	*Pseudozyma limtongiae*	SDBR-CMU686	PV834786
22	*Pseudozyma parantarctica*	JCM11752	AB089356
23	*Pseudozyma pollinicola*	SDBR-CMU619	PV834773
24	*Pseudozyma prolifica*	JCM10319	AB089368
25	*Pseudozyma pruni*	BCRC34227	EU379942
26	*Pseudozyma rugulosa*	JCM10323	AB089370
27	*Pseudozyma saisamorniae*	SDBR-CMU564	PV834778
28	*Pseudozyma shanxiensis*	SH64	DQ008956
29	*Pseudozyma siamensis*	DMS17137	AB117963
30	*Pseudozyma thailandica*	M9933	AB089354
31	*Pseudozyma tsukubaensis*	JCM10324	AB089372
32	*Sporisorium consanguineum*	H.U.V.19145	AY740048

33	*Sporisorium cordobense*	MP2634	AY740155
34	*Sporisorium elionuri*	MP2601	AY740157
35	*Sporisorium holwayi*	MP1271	AY344980
36	*Sporisorium modestum*	M56617	AY740054
37	*Sporisorium sorghi*	MP2036a	AY740021
38	*Sporisorium trachypogonis-plumosi*	M56635	AY740060
39	*Sporisorium veracruzianum*	MP735	AY747075
40	*Trichocintractia utriculicola*	HUV19.316	DQ875354
41	*Ustilago bouriqueti*	MS315	AY740167
42	*Ustilago calamagrostidis*	M56518	AY740065
43	*Ustilago hordei*	Ust.exs.784	AY345003

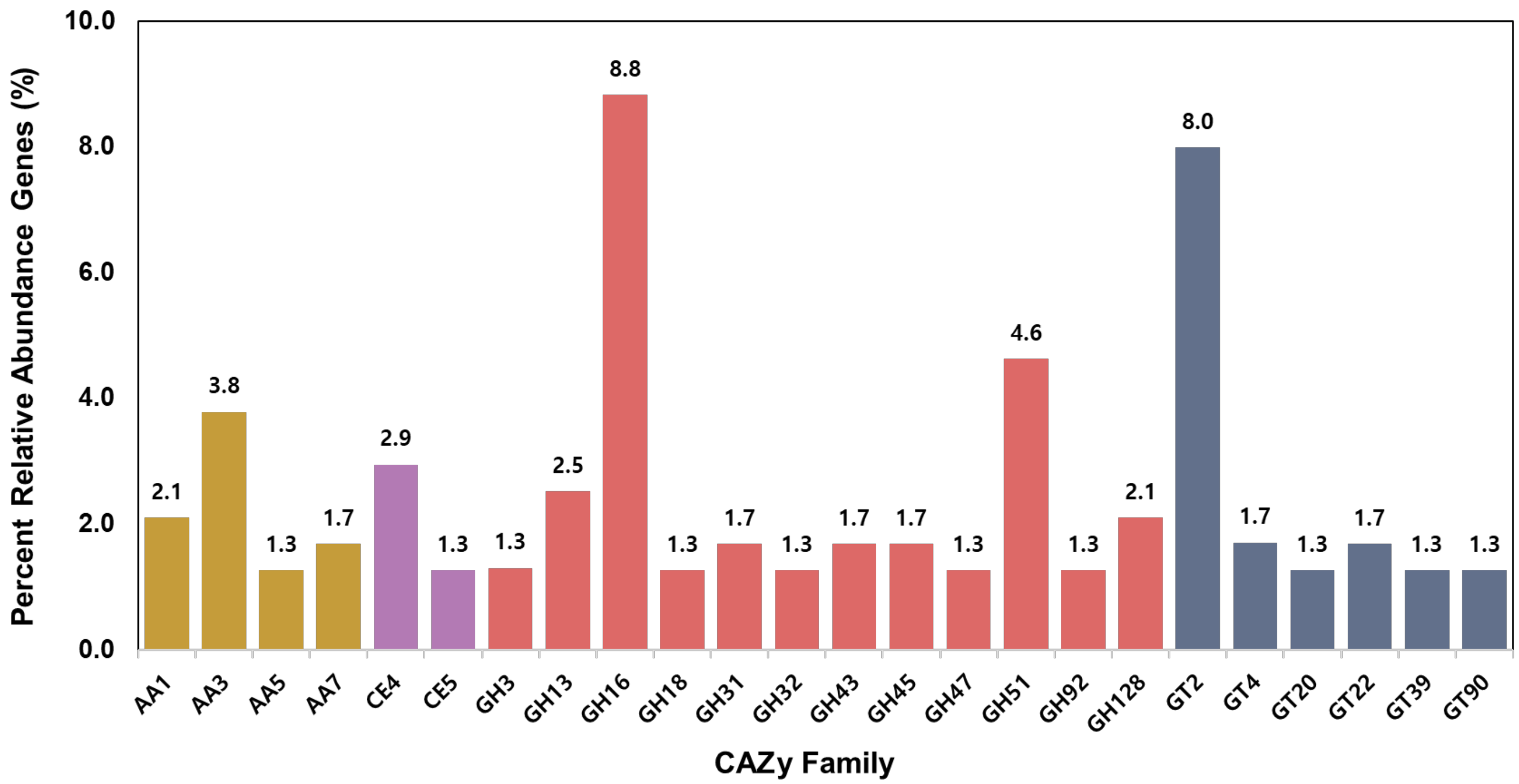

Figure S5. CAZy family distribution in Pseudozyma sp. SY16. CAZyme annotations identified by at least two of three approaches, HMMER, DIAMOND, and dbCAN-sub, were retained. Only families representing a relative abundance of 1.26% or greater were included. [AA1] Multicopper oxidase/laccase, [AA3] Glucose-methanol-choline oxidoreductases, [AA5] Copper radical oxidases, [AA7] Glucooligosaccharide oxidases, [CE4] Acetyl xylan esterases, chitin/ peptidoglycan deacetylases, [CE5] Acetyl xylan esterase, [GH3] β-glucosidase/β-xylosidase, [GH13] α-1,3-glucan synthases, [GH16] endo-1,3(4)-β-glucanases, [GH18] Chitinases, [GH31] α-glucosidases, α-galactosidases, [GH32] endo-inulinases, fructan β-fructosidases, [GH43] β-xylosidase, α-L-arabinofuranosidase, [GH45] Endoglucanase/cellulase, [GH47] α-mannosidase, [GH51] α-L-arabinofuranosidase, [GH92] α-mannosidase, [GH128] β-glucanase, [GT2] cellulose synthase, chitin synthase, [GT4] Glycogen/starch synthase, glycosyltransferase, [GT20] α,α-trehalose-phosphate synthase, [GT22] Glycosyltransferase, [GT39] Glycosyltransferase, [GT90] glucuronoxylomannan/galactoxylomannan β-1,2-xylosyltransferase.

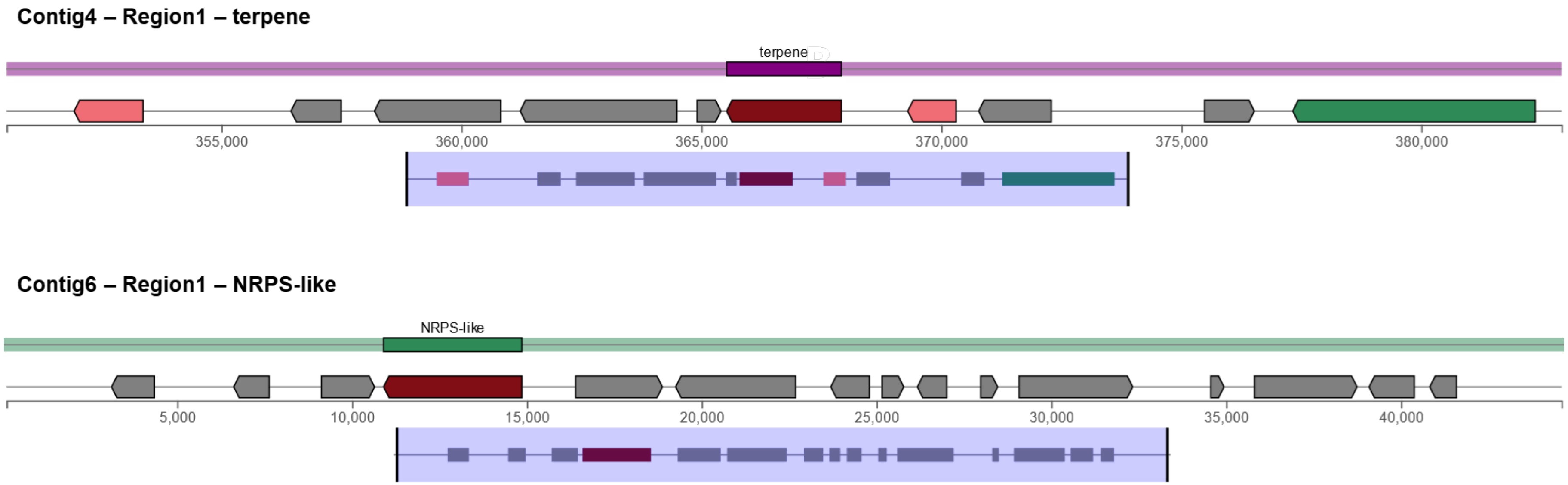

Figure S6. antiSMASH-predicted secondary metabolite biosynthetic gene clusters in *Pseudozyma* sp. SY16. Schematic representation of the terpene biosynthetic gene cluster identified on Contig4 and the NRPS-like biosynthetic gene cluster identified on Contig6. The Contig4 terpene cluster showed 97% similarity to the previously characterized clavaric acid biosynthetic gene cluster, whereas the Contig6 NRPS-like cluster showed 100% similarity to a previously reported choline-associated biosynthetic locus.

Table S2. Summary of predicted mating-type loci in *Pseudozyma* sp. SY16 and their sequence orthology.

Gene ID	Location (Scaffold: Range)	Description / Predicted Function	Top Hit Accession	Query Coverage (%)	Identity (%)	E-value	Bit Score
LOCUS_000613-RA	Contig1: 2–369	CDI51498.1 *b* mating type locus, *bE1* allele [*Melanopsichium pennsylvanicum*]	CDI51498.1	99.73	58	2E-123	373
LOCUS_000612-RA	Contig1: 1–425	CAA79219.1 Uh*bW1* [*Ustilago hordei*] CAJ42018.1 Uh*bW1* protein [*Ustilago hordei*]	CAA79219.1	83.33	46	6E-109	347
LOCUS_006065-RA	Contig19: 1–1254	CDS01535.1 hypothetical protein [*Sporisorium scitamineum*] CDU23481.1 related to PRM10-Pheromone-regulated protein, proposed to be involved in mating [*Sporisorium scitamineum*]	CDS01535.1	100	81	0.0	2004
